# Supplementary material for: Kangaroo mother care knowledge, attitude, and practice among nursing staff in a hospital in Jakarta, Indonesia
Source: PLoS One. 2021 Jun 4;16(6):e0252704. doi: 10.1371/journal.pone.0252704 (PMC8177461; doi:10.1371/journal.pone.0252704)
Supplement: Attachment — Submitted filename: Response to the reviewer 2.docx [file pone.0252704.s002.docx]

Response to the reviewer

Reviewer #2: I would like to thank the authors for their work in answering reviewers' questions.
I think the paper still has some minor issues that should be revised, namely inconsistencies in showing the results in the tables. In table 2 is still missing the row for n(%) as shown in table 1. In table 3 authors reported the p values inconsistently - with and without the 0 before the comma. I think authors should revise all tables again in order to present the results in a consistent way.

Our response:

We would like to thank the reviewer for giving the input for the manuscript. In the revised manuscript:

- We added n (%) in table 2
- We edited p-values in table 3 by adding 0 before the comma
- We revised the tables’ layout to make it consistent and easier to understand
